# Dendrobine Inhibits γ-Irradiation-Induced Cancer Cell Migration, Invasion and Metastasis in Non-Small Cell Lung Cancer Cells

**DOI:** 10.3390/biomedicines9080954

**Published:** 2021-08-03

**Authors:** Ye-Ram Kim, Ah-Reum Han, Jin-Baek Kim, Chan-Hun Jung

**Affiliations:** 1 Advanced Radiation Technology Institute, Korea Atomic Energy Research Institute, Jeongeup-si 56212, Jeollabuk-do, Korea; yrkim327@kaeri.re.kr (Y.-R.K.); arhan@kaeri.re.kr (A.-R.H.); jbkim74@kaeri.re.kr (J.-B.K.); 2 Jeonju AgroBio-Materials Institute, Jeongu-si 54810, Jeolabuk-do, Korea

**Keywords:** dendrobine, ionizing radiation, non-small cell lung carcinoma, metastasis, animal model

## Abstract

The use of ionizing radiation (IR) during radiotherapy can induce malignant effects, such as metastasis, which contribute to poor prognoses in lung cancer patients. Here, we explored the ability of dendrobine, a plant-derived alkaloid from *Dendrobium nobile*, to improve the efficacy of radiotherapy in non-small cell lung cancer (NSCLC). We employed Western blotting, quantitative real-time (qRT)-PCR, transwell migration assays, and wound-healing assays to determine the effects of dendrobine on the migration and invasion of A549 lung cancer cells in vitro. Dendrobine (5 mm) inhibited γ-irradiation-induced migration and invasion of A549 cells by suppressing sulfatase2 (SULF2) expression, thus inhibiting IR-induced signaling. To investigate the inhibitory effects of dendrobine in vivo, we established a mouse model of IR-induced metastasis by injecting BALB/c nude mice with γ-irradiated A549 cells via the tail vein. As expected, injection with γ-irradiated cells increased the number of pulmonary metastatic nodules in mice (0 Gy/DPBS, 9.8 ± 1.77; 2 Gy/DPBS, 20.87 ± 1.42), which was significantly reduced with dendrobine treatment (2 Gy/Dendrobine, 10.87 ± 0.71), by prevention of IR-induced signaling. Together, these findings demonstrate that dendrobine exerts inhibitory effects against γ-irradiation-induced invasion and metastasis in NSCLC cells in vitro and in vivo at non cytotoxic concentrations. Thus, dendrobine could serve as a therapeutic enhancer to overcome the malignant effects of radiation therapy in patients with NSCLC.

## 1. Introduction

Lung cancer is the most common cause of cancer-related death, due to its high rate of metastasis [1]. Approximately 15% of lung cancer cases consist of small-cell lung cancer, while the remaining 85% of cases are non-small cell lung cancer (NSCLC). Despite recent improvements in diagnostic technologies, approximately 70% patients with NSCLC are diagnosed at an advanced stage with metastatic disease [2]. Among the various treatment options for NSCLC, including surgical resection, radiotherapy, chemotherapy, targeted therapy, and immunotherapy, radiotherapy with localized application is considered the most effective treatment for inoperable NSCLC [3].

During radiotherapy, patients are exposed to ionizing radiation (IR), such as γ-irradiation, which directly or indirectly kills cancer cells by inducing DNA damage and the production of reactive oxygen species (ROS) [4]. Unfortunately, studies have reported that IR can induce cancer cell migration and invasion in multiple types of cancer cell and animal models [5,6,7]. These IR-induced properties can cause malignant effects, such as local recurrence and distant metastasis, by increasing metastatic potential, leading to poor prognoses in cancer patients [8]. Therefore, new strategies that inhibit IR-induced cancer cell migration, invasion, and metastasis are needed to improve the therapeutic effects of IR.

Heparin sulfate proteoglycans (HSPGs) are glycosylated proteins found on the cell surface and within the extracellular matrix that are related to multiple activities, including tumor development. HSPG activity depends on heparin-binding protein ligands, such as cytokines, chemokines, morphogens, angiogenic factors, and growth factors, which are found at the cell surface [9,10]. The extracellular sulfatases (SULFs), SULF1 and SULF2, regulate various signaling pathways by modifying sulfate to disulfate at the 6-O-position within HSPGs. Studies have identified SULF2 as an oncogenic effector whose upregulation is associated with poor prognoses in patients with various cancers [9,11,12,13]. Moreover, SULF2 reportedly plays a key role in IR-induced migration and invasion in NSCLC [14]. Although the roles of SULF2 in cancer development have been studied extensively, inhibitors targeting SULF2 remain less explored. SULF2 could serve as a target to overcome the malignant effects of IR in NSCLC.

**Figure 1:**
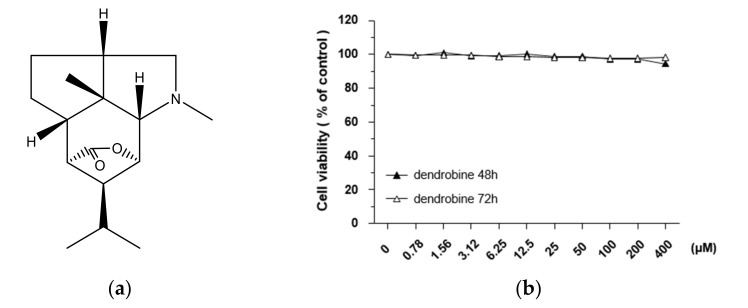
Effect of dendrobine on the viability of A549 lung cancer cells. (**a**) Chemical structure of dendrobine extracted from *Dendrobium nobile*. (**b**) Cell viability was measured using a Cell Counting Kit-8 assay kit after treatment with the indicated concentration of dendrobine for 48 or 72 h. Values are expressed as the mean ± SD of three independent experiments.

Dendrobine, a sesquiterpene alkaloid has been reported to be isolated from the medicinal plant *Dendrobium nobile* in a yield of 0.5% by dry weight (Figure 1a) [15]. This compound has been shown to exert various pharmacological activities, such as anti-pyretic and analgesic effects [16], neurotransmission effects through amino acid antagonism and presynaptic inhibition [17], and preventive effects on apoptosis and synaptic loss in neurons by amyloid *β* [18]. Metabolic studies have also reported that dendrobine is present at higher concentrations in the liver than in other tissues, suggesting that it may exert protective effects in the liver [19]. Moreover, recent biological studies have demonstrated that dendrobine possesses an antiviral activity against influenza A viruses during the early stage of the viral replication cycle [20] and hepatoprotective effects against carbon tetrachloride-induced mouse liver injury by activating the antioxidant nuclear factor erythroid 2-related factor 2 (Nrf2) pathway [19]. Furthermore, dendrobine alleviates gestational diabetes mellitus symptoms and inflammation in mice via T helper 17 (Th17) cells [21] and enhances the anticancer activity of cisplatin against human NSCLC A549 cells in vitro and in vivo [22]. However, the effects of dendrobine against IR-induced cancer cell migration, invasion, and metastasis, as well as its molecular mechanisms, have not yet been investigated.

In this study, we evaluated the anti-migratory and -invasion activities of dendrobine and its associated mechanisms in γ-irradiated A549 cells and in a mouse model of IR-induced metastasis that was established using tail-vein injection of γ-irradiated A549 lung cancer cells. Together, our findings demonstrate the potential of dendrobine as a lung cancer radiotherapy enhancer and also provide a reference for the design of a mouse model of IR-induced cancer metastasis.

## 2. Materials and Methods

### 2.1. Antibodies and Chemicals

The following antibodies were used in this study: anti-β-catenin (Santa Cruz Biotechnology; Santa Cruz, CA, USA), anti-phospho-STAT3 (Cell Signaling Technology; Danvers, MA, USA), anti-STAT3 (Cell Signaling Technology), anti-Bcl-X_L_, anti-phospho-Src (Cell Signaling Technology), anti-Src (Cell Signaling Technology), and anti-β-actin (Sigma-Aldrich; St. Louis, MO, USA). Dendrobine was purchased from Wuhan ChemFaces Biochemical (Hubei, China), and its purity (98%) and structure were confirmed using ^1^H NMR analysis (JNM-ECA 500 MHz NMR instrument, JEOL Ltd., Tokyo, Japan).

### 2.2. Cell Culture and Treatment

A549 lung cancer cells were purchased form the Korean Cell Line Bank (Seoul, Korea) and cultured in RPMI-1640 medium (Hyclone, Logan, UT, USA) supplemented with 10% FBS (Hyclone) at 37 °C in a humidified atmosphere with 5% CO_2_. For irradiation, A549 cells were seeded in a 60 mm dish at a density of 5 × 10^5^ cells, grown until they reached 70–80% confluence, and exposed to 10 Gy of γ-irradiation using a 137 Cs γ-ray source (Atomic Energy of Canada, Mississauga, Canada). The cells were then incubated with or without 5 µm dendrobine for 24 h and harvested for further studies.

### 2.3. Cell Viability Assay

Cell viability was measured using a Cell Counting Kit-8 (CCK-8) assay kit (Dojindo, Kumamoto, Japan), according to the manufacturer’s instructions. Briefly, cells were seeded in 96-well plates at a density of 2 × 10^3^ cells per well. Cells in each well were treated with various concentrations of dendrobine (0.78–400 µm) for 48 or 72 h and then incubated with 10 μL CCK-8 reagent for 4 h. Absorbance was measured at 450 nm using a VICTOR3 1420 Multilabel Counter (PerkinElmer, Waltham, MA, USA).

### 2.4. Wound-Healing Assay

Cell migration was analyzed using a CytoSelect^TM^ 24-Well Wound Healing Assay Kit (Cell Biolabs, San Diego, CA, USA), according to the manufacturer’s instructions. Briefly, A549 cells were seeded at a density of 3.5 × 10^5^ cells per well in 24-well plates containing plastic inserts to generate a wound area. After incubation for 24 h, the inserts were removed and the cultured cells were exposed to 10 Gy IR using a ^137^Cs γ-ray source (Atomic Energy of Canada), before being washed with medium and treated with 5 μm dendrobine. The cells were imaged at 0 and 24 h using an AE31 microscope connected to a Moticam-3+ digital camera (Motic, Hong Kong) at a total magnification of 12.5× (lens, 2.5×; objective, 10×; and camera, 0.5×).

### 2.5. Invasion Assay

Cell invasion was assessed using transwell assays, as described previously [23]. Briefly, A549 cells were seeded at a density of 1 × 10^4^ cells per well onto the upper surfaces of Matrigel-coated transwell chambers (BD Biosciences, Bedford, MA, USA) in serum-free medium. After treatment with 5 µm dendrobine for 16 h, cells that had invaded the lower surface of the chamber were stained using a Diff-Quick Kit (Fisher Scientific, Pittsburgh, PA, USA) and counted under an AE31 microscope.

### 2.6. Animal Study

All animal care and experimental procedures were approved by the Institutional Animal Care and Use Committee of Jeonbuk National University Hospital (Jeonju, Korea; cuh-IACUC-2019-23). The animal study protocol was performed in accordance with the guidelines and regulations set and approved by Jeonbuk National University Hospital.

### 2.7. IR-Induced Metastasis Mouse Model

Six-week-old female BALB/c nude mice (Orient Bio; Seongnam, Korea) were injected via the tail vein with A549 cells (1 × 10^6^ cell/mouse) previously incubated for 24 h and exposed to 0, 2, or 5 Gy IR. Five weeks later, the mice were euthanized, and their lungs were excised, imaged, and the number of metastasized tumors was counted. Tumor tissues were collected and subjected to histological or quantitative real-time (qRT)-PCR analyses.

### 2.8. A549 Xenograft Model

IR-exposed A549 cells (1 × 10^8^ cells/mL) in 10% Matrigel (Corning, NY, USA) were prepared, and then the IR-exposed A549 cells (1 × 10^6^ cells in 100 µL) were transplanted into the flank of six-week-old female BALB/c nude mice. When the tumor reached approximately 80–120 mm^3^, the mice were randomly divided into four treatment groups (DPBS; 1 mg/kg Dendrobine; 5 mg/kg Dendrobine; 25 mg/kg Dendrobine). The mice were intraperitoneally (ip) injected daily with dendrobine (1, 5, or 25 mg/kg/day) or dulbecco’s phosphate-buffered saline (DPBS) for two weeks. Tumors were measured twice weekly using a digital caliper, and tumor volume was calculated as follows: V (mean tumor volume) = (width^2^ × length)/2. Mouse body weight was also measured.

### 2.9. Metastasis Assay in the IR-Induced Metastasis Mouse Model

A549 cells were seeded and incubated for 24 h before being exposed to 2 Gy γ-irradiation using a TrueBeam instrument (Varian Medical Systems, Palo Alto, CA, USA) and incubated for 24 h. Six-week-old female BALB/c nude mice were then injected via the tail vein with non-irradiated and irradiated A549 cells (1 × 10^6^ cells/mouse) and divided into four groups (0 Gy/DPBS; 0 Gy/dendrobine; 2 Gy/DPBS; 2 Gy/dendrobine). Dendrobine (5 mg/kg/day) was administered daily to the indicated groups using ip injection for five weeks. Mice were then euthanized, and their lungs were excised, photographed, and the number of metastasized tumors was counted. Tumor tissues were collected and subjected to histological, Western blot, and qRT-PCR analysis.

### 2.10. Western Blotting

A549 cells and mouse lung tissues were lysed using radioimmunoprecipitation assay (RIPA) buffer (25 mm Tris-HCl pH 7.6, 150 mm NaCl, 1% NP-40, 1% sodium deoxycholate, and 0.1% sodium dodecyl sulfate (SDS)) containing a protease inhibitor cocktail (Roche, Basel, Switzerland). Equal amounts of protein lysates were separated using SDS-PAGE, electrotransferred onto nitrocellulose membranes (Millipore, Burlington, MA, USA), and analyzed using specific antibodies with an enhanced chemiluminescent (ECL) detection system (Bio-Rad, Hercules, CA, USA).

### 2.11. Enzyme-Linked Immunosorbent Assay (ELISA)

SULF2 protein levels in cell lysates were measured using a human SULF2/Sulfatase 2 ELISA kit (LSBio, Seattle, WA, USA), according to the manufacturer’s protocol.

### 2.12. qRT-PCR

Total RNA was isolated using a RNeasy Mini Kit (Qiagen, Valencia, CA, USA) and reverse-transcribed into cDNA using a PrimeScript 1st strand cDNA Synthesis Kit (Takara Bio, Kyoto, Japan), according to the instructions of the manufacturers. qRT-PCR was con-ducted using a CFX96 Touch Real-Time PCR Detection System (Bio-Rad) with TB Green Premix Ex Taq II (Takara Bio). The primer sequences are shown in Table 1. Data were normalized to the house-keeping gene GAPDH.

### 2.13. Statistical Analysis

in vitro experiments were performed as three independent experiments with at least three replicates to obtain data as the mean ± standard deviation (SD). Statistical significance was determined using one-way analysis of variance (ANOVA) in GraphPad Prism software (GraphPad Software, La Jolla, CA, USA). *p* values <0.05 were considered statistically significant. The in vivo data were analyzed using Levene’s test, followed by one-way ANOVA in GraphPad and Tukey’s post hoc test. The confidence interval was set to 95%. in vivo data are presented as the mean ± SD.

## 3. Results

### 3.1. Effects of Dendrobine on A549 Cell Viability

To investigate the cytotoxic effects of dendrobine (Figure 1a), we performed MTT assays on A549 lung cancer cells treated with various concentrations of dendrobine (0.78–400 µm) for 48 or 72 h. As shown in Figure 1b, dendrobine exerted no cytotoxic effects; therefore, further studies were performed using 5 µm dendrobine to test the effective concentration of dendrobine for inhibiting IR-induced malignant effects, while minimizing the potential for toxic effects on cells.

### 3.2. Inhibitory Effects of Dendrobine on IR-Induced Migration and Invasion in A549 Cells

**Figure 2:**
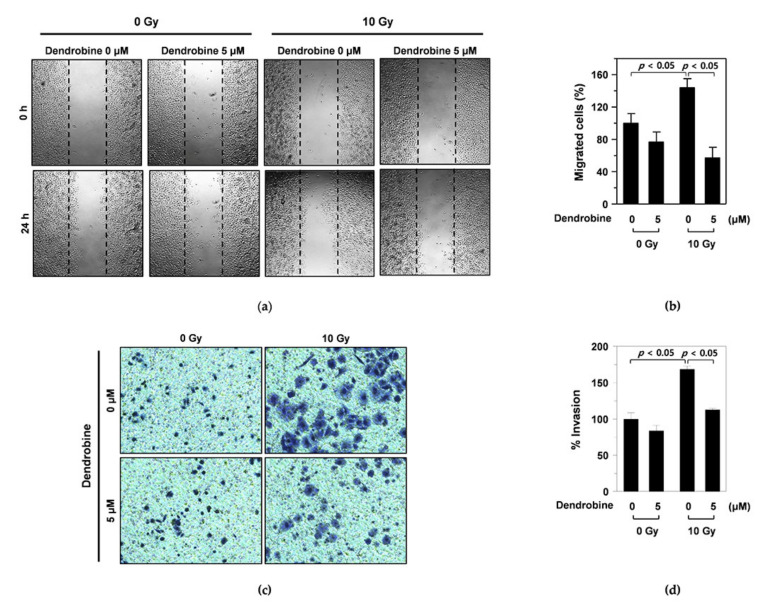
Inhibitory effect of dendrobine on IR-induced migration and invasion of A549 lung cancer cells. (**a**) The inhibitory activity of dendrobine on IR-induced migration was measured using a wound healing assay. (**b**) Quantification of the migrated cells. The relative migrated cells were calculated as the ratio of the migrated cells at a given time point at 0 h. Data represent the mean ± SD (*n* = 3). (**c**) The inhibitory activity of dendrobine on IR-induced invasion was determined using an invasion assay. (**d**) Quantification of invasion. The percentage of invasion is represented as the number of cells per field compared with the control (0 Gy/0 µm of dendrobine) group. Data represent the mean ± SD (*n* = 3).

To investigate whether dendrobine affected IR-induced migration in A549 cells, we performed wound-healing assays. As shown in Figure 2a, the width of the scratch created in A549 cells was rapidly recovered following 10 Gy of γ-irradiation; however, this recovery was reduced by treatment with 5 µm dendrobine, as confirmed by cell migration quantification (Figure 2b). Next, we performed invasion assays using a transwell chamber to test whether dendrobine also exerted inhibitory effects against IR-induced cancer cell invasion. As expected, 10 Gy of IR promoted the invasion of A549 cells, whereas this effect was decreased by treatment with 5 μm dendrobine (Figure 2c). This result was further confirmed by quantifying cancer cell invasion (Figure 2d). Together, these findings indicate that dendrobine inhibits IR-induced migration and invasion in A549 lung cancer cells.

### 3.3. Dendrobine Inhibits Ir-Induced Cancer Cell Migration and Invasion by Suppressing SULF2 mRNA Levels

**Figure 3:**
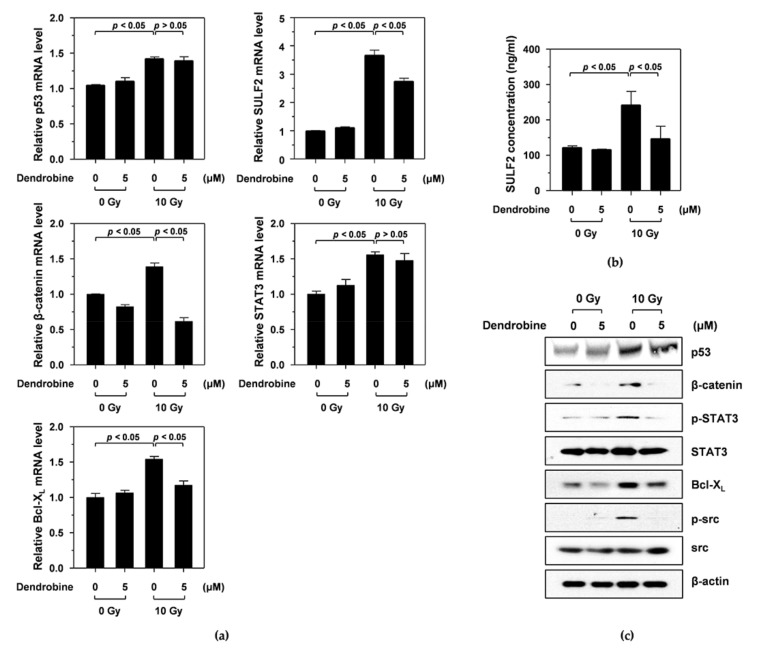
Inhibitory effect of dendrobine on IR-induced signaling, by inhibiting the mRNA and protein expression of SULF2. (**a**) mRNA levels of IR-induced signaling molecules, including p53, SULF2, β-catenin, STAT3, and Bcl-X_L_, were measured using qRT-PCR. (**b**) SULF2 protein expression was determined using an ELISA kit. (**c**) Protein expression levels of p53, β-catenin, phosphorylated STAT3, STAT3, Bcl-X_L_, phosphorylated Src, and Src were measured using Western blotting.

A previous study reported that IR promotes cancer cell migration and invasion by stimulating the SULF2/β-catenin/signal transducer and activator of transcription 3 (STAT3)/superoxide dismutase 2 (SOD2)/Bcl-X_L_ pathway via the p53 transcription factor [14]. To investigate whether dendrobine inhibited IR-induced signaling molecules, including p53, SULF2, β-catenin, STAT3, and Bcl-X_L_, we performed qRT-PCR. As shown in Figure 3a, IR induced an increase in p53 mRNA expression, which increased the mRNA levels of downstream signaling molecules, such as SULF2, β-catenin, STAT3, and Bcl-X_L_. Interestingly, treatment with 5 µm dendrobine decreased SULF2, β-catenin, and Bcl-X_L_ mRNA levels after IR but did not suppress p53 and STAT3.

Next, we analyzed the effects of dendrobine on the protein expression of IR-induced signaling molecules by performing ELISA and Western blot assays. Consistent with the qRT-PCR results, IR increased p53, SULF2, β-catenin, phosphorylated STAT3, and Bcl-X_L_ expression, all of which were significantly suppressed by 5 μm dendrobine, except for p53 (Figure 3c). A previous study also reported that the mitochondrial ROS produced by Bcl-X_L_ promoted cancer cell invasion via an Src-dependent pathway [24]. Similarly, we found that IR increased Src phosphorylation, which was abolished by 5 µm dendrobine (Figure 3c). Therefore, these results suggest that dendrobine inhibits IR-induced signaling by suppressing the mRNA and protein expression of SULF2, an upstream signaling molecule involved in the response to IR.

### 3.4. Establishment of the IR-Induced Metastasis Mouse Model

**Figure 4:**
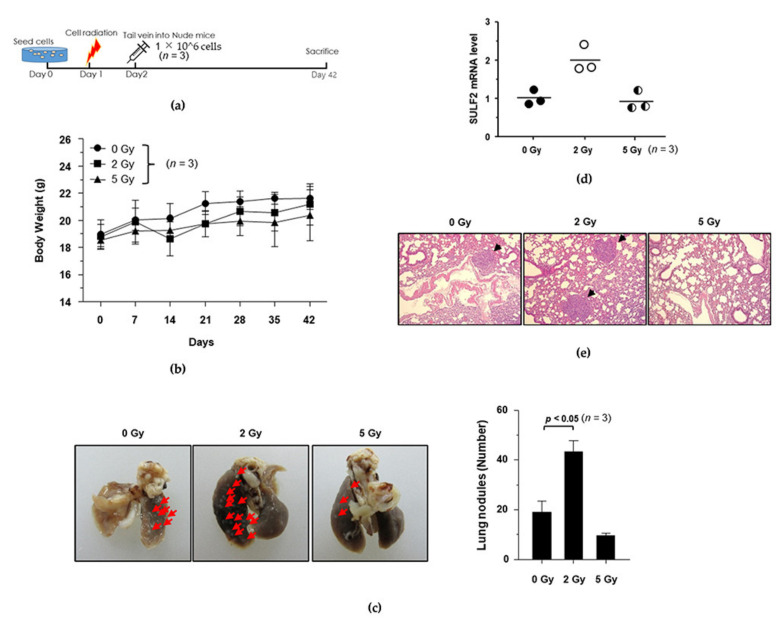
Establishment of a mouse model of IR-induced metastasis. (**a**) Schematic diagram of the experimental design for establishing the mouse model of IR-induced metastasis. (**b**) Changes in mouse body weights. (**c**) Representative gross lung images. Red arrows indicate metastatic nodules. (**d**) SULF2 mRNA levels in lung tissues were compared using qRT-PCR. (**e**) H&E staining of lung specimens. Black arrows indicated metastatic nodules.

To explore the inhibitory potential of dendrobine against IR-induced cancer cell migration and invasion in vivo, we established a mouse model of IR-induced metastasis. First, we determined the dose of radiation that was required to induce metastatic activity in vivo by irradiating A549 cells with γ-rays (0, 2, and 5 Gy). The irradiated and untreated control cells were injected into mice via the tail vein and the number of nodules in the lungs was measured (Figure 4a). As shown in Figure 4b, the body weight of the mice was not significantly affected upon injection of 2 or 5 Gy IR-exposed lung cancer cells, whereas the number of lung nodules in the lung was significantly increased by 2 Gy IR, but not 5 Gy, as confirmed by quantitative analysis (Figure 4c).

To investigate whether SULF2 mediates the formation of lung metastatic tumor nodules in vivo, we measured SULF2 mRNA levels in lung tissues using qRT-PCR. Interestingly, exposure to 2 Gy of IR significantly increased SULF2 mRNA levels, but 5 Gy of IR did not (Figure 4d). The number of metastatic nodules was confirmed using hematoxylin and eosin (H&E) staining, which revealed that 2 Gy of IR promotes the metastatic ability of A549 cells in vivo (Figure 4e). Therefore, we selected a dose of 2 Gy to investigate the inhibitory potential of dendrobine against IR-induced metastasis in the mouse model.

### 3.5. Effects of Dendrobine on Tumor Growth in the Xenograft Model

**Figure 5:**
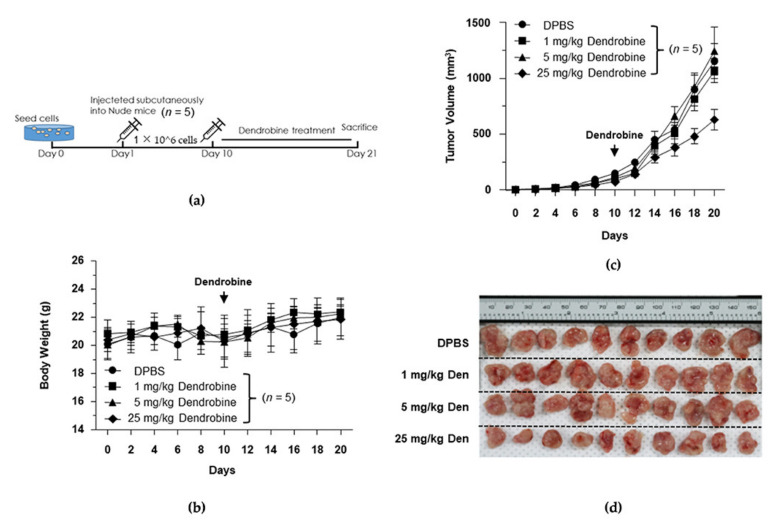
Inhibitory effects of dendrobine on tumor growth in a xenograft model. (**a**) Schematic diagram of the experimental design for investigating the inhibitory effects of dendrobine on tumor growth. (**b**) Changes in mouse body weights. (**c**) Changes in mouse tumor volumes. (**d**) Image of tumors isolated at the end of the experiment.

To investigate the effects of dendrobine on tumor growth in vivo, we subcutaneously injected BALB/c nude mice with A549 lung cancer cells. When the average tumor volume had reached ~100 mm^3^, the mice were i.p. injected with DPBS or dendrobine (1, 5, or 25 mg/kg) once a day for 14 days (Figure 5a). As shown in Figure 5b, no significant changes in body weight were observed between the four groups; however, the mean tumor volume was significantly lower in the 25 mg/kg dendrobine-treated mice than in the other groups (control, 1 and 5 mg/kg; Figure 5c,d). Together, these results suggest that dendrobine does not affect tumor growth at a concentration of 1 or 5 mg/kg. Therefore, we used a non-cytotoxic dendrobine dose (5 mg/kg) to further investigate its inhibitory potential against IR-induced metastasis in the established mouse model.

### 3.6. Anti-Metastasis Efficacy of Dendrobine in the Mouse Model of IR-Induced Metastasis

**Figure 6:**
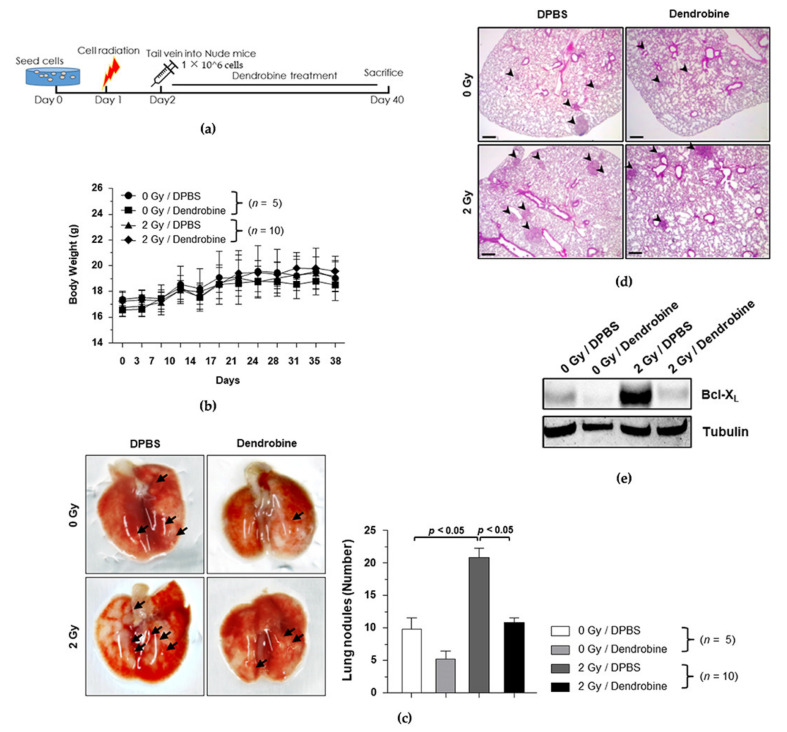
Inhibitory effects of dendrobine on IR-induced metastasis in vivo. (**a**) Schematic diagram of the experimental design for investigating the inhibitory effect of dendrobine on IR-induced metastasis. (**b**) Change in mouse body weights. (**c**) Representative gross lung images. Black arrows indicate metastatic nodules measured for quantitative analysis. (**d**) H&E staining of lung specimens. (**e**) Bcl-X_L_ protein levels in lung tissues were compared using Western blotting.

To investigate the anti-metastasis efficacy of dendrobine in vivo, we used the optimal IR dose of 2 Gy to induce cancer metastasis in the established metastasis mouse model, followed by treatment with a non-toxic concentration of dendrobine (5 mg/kg), according to the experimental schedule shown in Figure 6a. Body weight did not change significantly in any of the groups (Figure 6b); however, the number of metastatic nodules was significantly increased by exposure to 2 Gy of IR and markedly reduced by dendrobine (5 mg/kg) treatment (Figure 6c). To confirm these findings, lung tissues were collected and histologically analyzed using H&E staining (Figure 6d), while Western blotting was performed to explore whether dendrobine suppresses the expression of proteins in the IR-induced signaling pathway in vivo. As shown in Figure 6e, the protein levels of Bcl-X_L_ were increased after exposure to 2 Gy of IR, but significantly suppressed by dendrobine treatment. Taken together, these results suggest that dendrobine suppresses IR-induced metastasis in vivo by inhibiting the Bcl-X_L_ expression involved in SULF2-mediated signaling.

## 4. Discussion

Previously, we reported that sublethal doses of IR induce malignant effects, such as cell invasion, by stimulating the SULF2/β-catenin/STAT3/SOD2/Bcl-X_L_ signaling pathway via p53 in vitro and in vivo; whereas mitochondrial ROS produced by Bcl-X_L_ promote cancer cell invasion via an Src-dependent pathway [5,14,24]. Thus, the SULF2 pathway represents a potential therapeutic target for overcoming the malignant effects of IR. Since then, we have been continuously searching for novel radiotherapy enhancers derived from medicinal plants to improve the therapeutic efficacy of radiotherapy [24,25,26]. Among the compounds screened, dendrobine has shown a strong inhibitory activity against IR-induced cancer cell migration. Although dendrobine has been reported to exert various biological effects, few studies have investigated its anticancer effects. Recently, dendrobine was reported to act as a potential chemotoxicity sensitizer against A549 lung cancer cells alongside cisplatin treatment, by stimulating the JNK-mediated signaling pathways to enhance cisplatin-induced apoptosis in vitro and in vivo [22]. However, no studies have reported whether dendrobine enhances the therapeutic effects of radiotherapy in A549 lung cancer cells.

In this study, we investigated the potential efficacy of a radiotherapy enhancer using dendrobine, which is mainly found in *Dendrobium nobile*. First, we demonstrated that non-cytotoxic dendrobine concentrations inhibited IR-induced migration and invasion in A549 lung cancer cells (Figure 2). Next, we attempted to identify which IR-induced signaling molecules were inhibited by dendrobine; the results revealed that dendrobine significantly suppressed SULF2 mRNA and protein expression in vitro. Together with our previous findings, these results suggest that dendrobine inhibits IR-induced cancer cell migration and invasion by suppressing the mRNA and protein expression of SULF2, a key mediator of IR-induced signaling [14].

To clarify the inhibitory potential of dendrobine against IR-induced cancer cell migration and invasion in vivo, we established a mouse model of IR-induced metastasis. A previous study revealed that exposure to 2 Gy IR did not significantly affect the growth of H460 lung cancer cells in a xenograft model but promoted SULF2 mRNA levels [14]. Consistently, we found that exposure to 2 Gy IR, not 5 Gy, also promoted SULF2 mRNA expression in our lung metastasis model injected with A549 lung cancer cells via the tail vein. Therefore, we selected a dose of 2 Gy IR for our metastatic mouse model, to investigate the inhibitory effects of dendrobine in vivo. Given that dendrobine showed cytotoxicity at 50 mg/kg in xenograft tumor growth [22], it may also show cytotoxicity at concentrations lower than 50 mg/kg. Therefore, we evaluated the cytotoxicity at 1, 5, and 25 mg/kg concentrations of dendrobine to measure its effect on tumor growth in a xenograft tumor model; since cytotoxicity would limit its efficacy as a radiotherapeutic enhancer. Neither 1 nor 5 mg/kg dendrobine affected tumor growth; therefore, we evaluated the inhibitory potential of a 5 mg/kg dose of dendrobine against IR-induced metastasis in the established mouse model. We found that 5 mg/kg dendrobine exerted anti-metastatic effects by inhibiting the Bcl-X_L_ expression involved in SULF2-mediated signaling, preventing IR-induced signaling. Together, these findings suggest that dendrobine can be used as a radiotherapy efficiency enhancer, capable of significantly inhibiting IR-induced cancer cell invasion and metastasis by suppressing SULF2 mRNA and protein expression in vitro and in vivo.

Some studies have reported that dendrobine exhibits anticancer activities in various human cancers, including ovarian cancer, promyelocytic leukemia, sarcoma, and lung cancer, and inhibits tumor growth in xenograft models [22,27,28,29,30]. Consistently, we observed that dendrobine inhibited tumor growth at doses greater than 25 mg/kg and also inhibited IR-induced migration and invasion in vitro and IR-induced metastasis in vivo at doses lower than 5 mg/kg. Therefore, dendrobine may improve anticancer therapies by both restraining tumor growth and acting as a therapeutic enhancer to prevent the malignant effects of IR.

Although novel effective cancer treatments have been developed, such as immunotherapy [30,31], traditional cancer therapies such as chemotherapy and radiotherapy remain widely used. Therefore, it is necessary to develop new strategies to improve the therapeutic effects of traditional treatments. Here, we demonstrated that dendrobine could act as a therapeutic enhancer by preventing SULF2 expression during radiotherapy. Consistently, previous studies have suggested that dendrobine could be used as a chemosensitizer for cisplatin to improve its anticancer activities by inducing cell death and inhibiting tumor growth [22]. Although further studies are required to determine the optimal concentration of dendrobine for application as a chemosensitizer and radiotherapy enhancer, dendrobine displays potential as a novel therapeutic enhancer to overcome the limitations and improve the effects of existing treatments for NSCLC.

## 5. Conclusions

In conclusion, the findings of this study suggest that dendrobine treatment effectively inhibits IR-induced NSCLC cell migration and invasion under non-toxic conditions. Moreover, we demonstrated that these effects are mediated by dendrobine, which suppresses the mRNA expression of SULF2, a key mediator of IR-induced malignant effects, in vitro and in vivo. Thus, dendrobine could serve as a potential therapeutic enhancer, to overcome the malignant effects of IR in NSCLC.

## Figures and Tables

**Table 1:** Primer sequences used for qPCR.

Gene	Sequence (5′-3′)	
*p53*	Forward	CAT GAG CGC TGC TCA GAT AG
	Reverse	TGG TAC AGT CAG AGC CAA CCT
*SULF2*	Forward	TGT CAT TGT CTC TCT TGT GTA GC
	Reverse	AAT CCA TCC TCA AGC TGC TG
*β-catenin*	Forward	GTC CTC TGT GAA CTT GCT CAG
	Reverse	CCT CAG ACA TTC GGA ACA AAA
*STAT3*	Forward	CCC CGC ACT TTA GAT TCA TTG
	Reverse	AGG TCA ACT CCA TGT CAA AGG
*GAPDH*	Forward	ACT CCA CTC ACG GCA AAT TC
	Reverse	TCT CCA TGG TGG TGA AGA CA
